# *Citrus limon*-derived nanovesicles inhibit cancer cell proliferation and suppress CML xenograft growth by inducing TRAIL-mediated cell death

**DOI:** 10.18632/oncotarget.4004

**Published:** 2015-05-18

**Authors:** Stefania Raimondo, Flores Naselli, Simona Fontana, Francesca Monteleone, Alessia Lo Dico, Laura Saieva, Giovanni Zito, Anna Flugy, Mauro Manno, Maria Antonietta Di Bella, Giacomo De Leo, Riccardo Alessandro

**Affiliations:** ^1^ Dipartimento di Biopatologia e Biotecnologie Mediche, Università degli Studi di Palermo, sezione di Biologia e Genetica, Palermo, Italy; ^2^ Laboratorio di Ingegneria Tissutale – Piattaforme Innovative per l′Ingegneria Tissutale (PON01–00829), Istituto Ortopedico Rizzoli, Palermo, Italy; ^3^ Istituto di Biofisica, Consiglio Nazionale delle Ricerche, Palermo, Italy

**Keywords:** cancer, exosome-like nanovesicles, *Citrus limon* L., TRAIL-mediated cell death

## Abstract

Nanosized vesicles are considered key players in cell to cell communication, thus influencing physiological and pathological processes, including cancer. Nanovesicles have also been found in edible-plants and have shown therapeutic activity in inflammatory bowel diseases; however information on their role in affecting cancer progression is missing.

Our study identify for the first time a fraction of vesicles from lemon juice (*Citrus limon* L.), obtained as a result of different ultracentrifugation, with density ranging from 1,15 to 1,19 g/ml and specific proteomic profile. By using an *in vitro* approach, we show that isolated nanovesicles inhibit cancer cell proliferation in different tumor cell lines, by activating a TRAIL-mediated apoptotic cell death. Furthermore, we demonstrate that lemon nanovesicles suppress CML tumor growth *in vivo* by specifically reaching tumor site and by activating TRAIL-mediated apoptotic cell processes. Overall, this study suggests the possible use of plant-edible nanovesicles as a feasible approach in cancer treatment.

## INTRODUCTION

Physiological cell to cell communication occurs in order to maintain tissue homeostasis. Among the different mechanisms that have been described in the past years, extracellular vesicle-mediated cell interaction has attracted recently the interest of researchers because of the ability of these vesicles to shuttle a variety of molecules from the producing cell to target cells [1]. Extracellular vesicles (EVs) are membranous vesicles of different size (30–1000 nm), released by a variety of cell types. Among the EVs different types, exosomes are nanometer sized vesicles (30–100 nm) present in biological fluids of different organisms. They carry various molecular constituents of the producing cell, including proteins, lipids, mRNAs, and microRNAs (miRNAs) [1]. An increasing number of evidences have demonstrated that exosomes exert an important role in cell-to-cell communication and influence both physiological and pathological processes, such as cancer and neurodegenerative disorders [1–4]. Additionally, molecular constituents in exosomes have been found to be associated with certain diseases and treatment responses, indicating that they may also serve as a diagnostic tool [5].

Previous studies suggested that nanosized particles from plant cells may be exosome-like [6]. Zhang and colleagues have reported that nanoparticles derived from edible plants (grape, grapefruit, ginger and carrots) show anti-inflammatory properties in inflammatory bowel diseases [7, 8]. Although it has been shown that compounds and/or aqueous extracts from different plant varieties exert anti-proliferative and anticancer activity [9–12], the specific role of plant-derived nanovesicles to influence cancer progression is still unknown.

The tumor necrosis factor (TNF)-related apoptosis-inducing ligand-receptor (TRAIL-R) family has emerged as a key mediator of cell fate and survival, by initiating the extrinsic apoptotic pathway [13]. Importantly, unlike many chemotherapeutic drugs, TRAIL has the ability to induce apoptosis in transformed but not in normal cells, thus being considered of great therapeutic potential [14, 15]. In addition, most cancer cells can be sensitized for TRAIL-induced apoptosis [16].

Here, we show that the juice of *Citrus Limon* L. (family Rutaceae) contains nanoparticles, with morphological, dimensional and proteomic profile that allowed us to consider them as exosome-like nanovesicles. We found that isolated nanovesicles have *in vitro* antineoplastic activity on a panel of different solid and hematological cancers cell lines. Strikingly, we demonstrated that lemon-derived nanovesicles have also an effect *in vivo*, by suppressing the growth of a CML xenograft model. Moreover, we showed that lemon-derived nanoparticles exert their anticancer activity, by stimulating a TRAIL-mediated apoptotic mechanism.

All together, these findings highlight an alternative approach for cancer treatment, focused on using nanoparticles from natural substances, thus suggesting that the combination of natural agents and chemotherapy could be in the next future a feasible approach to eradicate cancer.

## RESULTS

### Isolation and characterization of *Citrus limon* L.-derived nanovesicles

**Figure 1:**
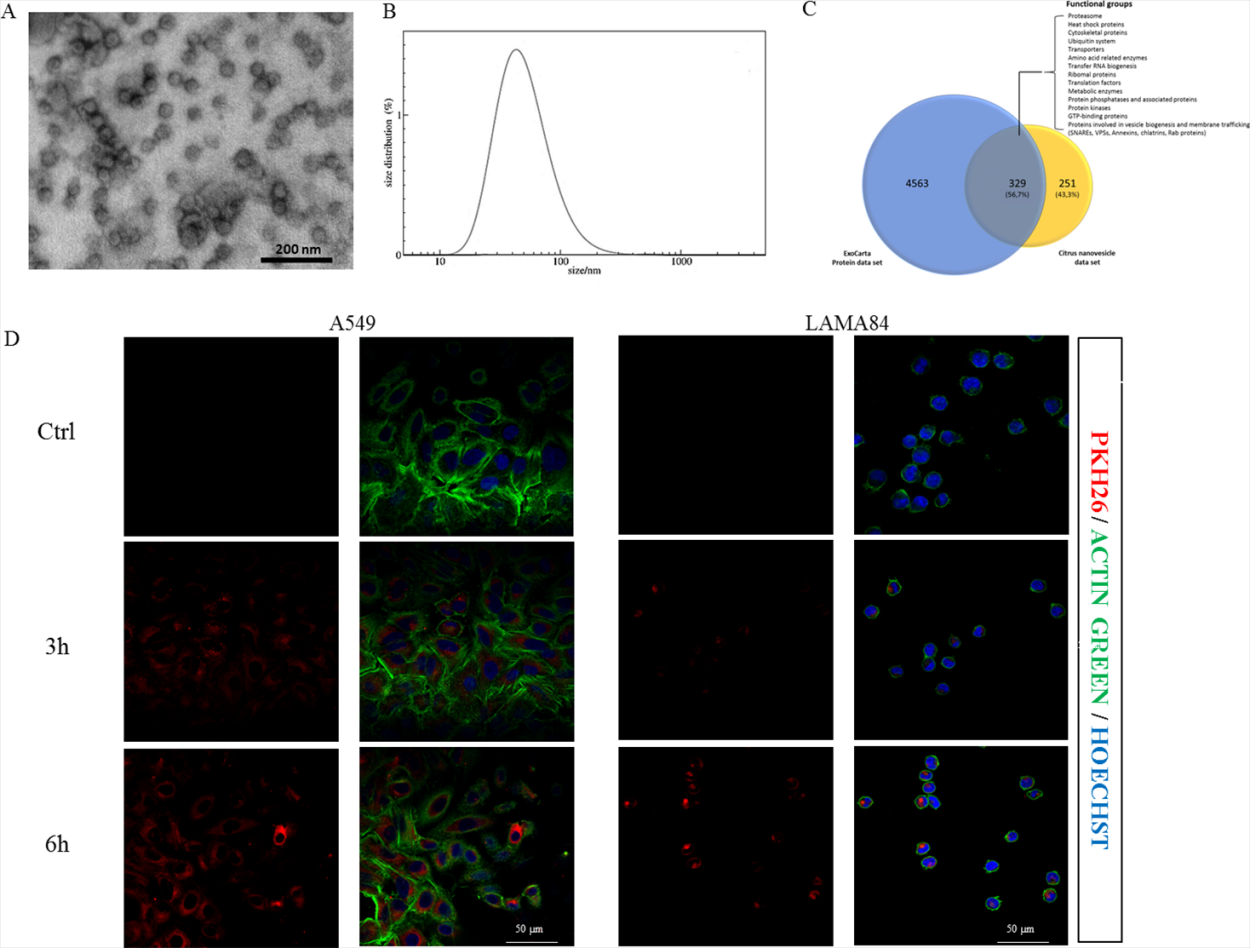
Nanovesicles characterization and uptake by target cells **A.** Citrus nanovesicles were collected after 30% sucrose gradient ultracentrifugation and analyzed at transmission electron microscope. The scale bar indicates 200 nm. **B.** Nanovesicles size distribution was determined by DLS analysis. **C.** The Venn diagram shows a comparison between the *Citrus* nanovesicle data set and protein ExoCarta data set. The overlapping area contains about 60% of proteins identified in *Citrus* nanovesicles. Most of these common proteins belong to functional groups highly associated with exosomes. **D.** Analysis at confocal microscopy of A549 (left panel) or LAMA84 cells (right panel) treated, for 3 and 6 hours, with 20 μg/ml of *Citrus* nanovesicles, compared with untreated cells (Ctrl). Cells were stained with Actin Green 488 (green), nuclear counterstaining was performed using Hoechst (blue), nanovesicles were labeled with PKH26 (red).

*Citrus limon* L. nanovesicles were isolated from the fruit juice using ultracentrifugation method and purification on a 30% sucrose gradient. Electron microscope analysis showed the integrity and size of isolated vesicles, ranged between 50–70 nm (Figure 1A). Nanovesicle size distribution was also confirmed by dynamic light scattering (DLS) experiments as shown in Figure 1B. Taken together, our data showed that nanovesicles identified in *Citrus limon* are exosome-like, based on their morphology and size.

### Proteome profiling of *Citrus limon juice* L.-derived nanovesicles

*Citrus limon* is a nonmodel plant species and due to the lack of complete genomic sequences and proteomic data, the availability of protein sequences in commonly employed databases is limited. Thus, to obtain maximum proteome coverage, it is usually suggested to perform a homology search by employing multiple databases. However, this strategy may determine a large number of identifications that generally are highly redundant and need to be checked, greatly affecting proteomic characterization and hindering a comprehensive and reliable protein identification. We therefore performed our search against a restricted protein database, namely *Citrus* database, which comprises 39096 entries (July 2014). By merging the results of GeLC-MS/MS and LC-MS/MS analyses, we confidently identified 580 proteins with a FDR of less than 1%. However, although confidently identified, many proteins were still uncharacterized from a functional point of view. In order to attribute a molecular function also to the “uncharacterized proteins”, we looked for all of identified proteins in KEGG ORTHOLOGY (KO) database that collects functionally identical genes (orthologs). The list of total proteins identified in *Citrus* nanoparticles is provided in Supplementary Table S1. In this table, for each identified protein we reported the results obtained by searching in both *Citrus* and KO databases, and numbers and sequences of corresponding peptides used for the identification. Finally, by comparing the *Citrus*-derived nanovesicles protein dataset with the exosome protein one reported in ExoCarta database (4563 entries), we found that 56.7% of proteins of our dataset overlapped with those previously identified as exosome proteins in mammalian tissues and cell types and belonging to functional groups that characterize exosomes regardless of their cellular origin (Figure 1C) [17].

### *Citrus limon* L.-derived nanovesicles are internalized and reduce the viability of cancer cells

In order to determine if C*itrus* nanovesicles are internalized by human cancer cells, nanovesicles were labeled with the lipophilic dye PKH26. The human lung carcinoma cell line A549 and the chronic myeloid leukemia cell line LAMA84 cells treated at 37°C with 20 μg/ml of nanovesicles for 3 or 6 h internalized lemon nanovesicles in a time dependent manner as shown in Figure 1D (left and right panel); the uptake was impaired after incubation at 4°C (Supplementary Figure 1A), thus confirming that nanovesicles uptake was mediated by a biologically active process.

**Figure 2:**
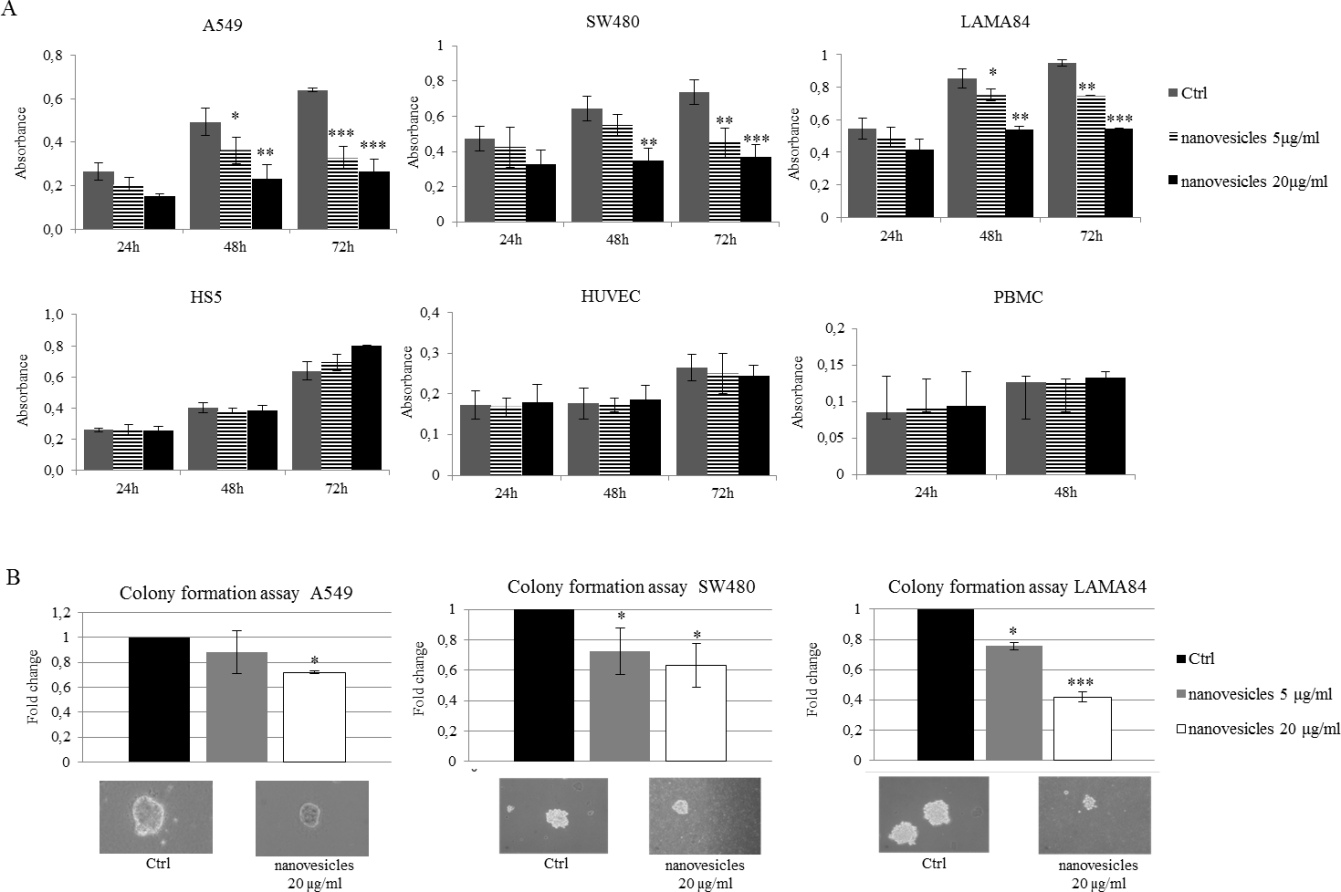
*Citrus* nanovesicles inhibit the growth of tumor cell lines **A.** Cell growth was measured by MTT assay after 24, 48, 72 h of treatment with 5 or 20 μg/ml of nanovesicles. The values were plotted as absorbance. Each point represents the mean ± SD of three independent experiments. **B.** Cancer cell survival was assessed by colony formation assay in methylcellulose. Cells were plated in methylcellulose in presence or not of 5 and 20 μg/ml of *Citrus* nanovesicles. The values were plotted as fold change compared to control cells (untreated cells). Each point in the histogram represents the mean ± SD of three independent experiments. Asterisks indicate statistically significant values in comparison to control (Ctrl) (*p ≤ 0.05; **p ≤ 0.01; ***p ≤ 0.001). Pictures are representative of observed colonies.

In order to test the ability of *Citrus*-derived nanovesicles to influence the growth of tumor cells, A549, SW480 (human colorectal adenocarcinoma cell line) and LAMA84 cells were treated for 24, 48 or 72 h with 5 or 20 μg/ml of nanovesicles. The MTT viability assay showed that lemon nanovesicles inhibited tumor cell viability in a dose and time dependent manner compared with untreated cells (Figure 2A upper panel). The results herein showed a 50% growth reduction of the three cell lines with 20 μg/ml of nanovesicles at 48 h time point. In order to assess the specificity of lemon nanovesicles against tumor cell lines, non-cancer cell lines HS5 (Human bone marrow stromal cells), HUVEC (Human Umbilical Vein Endothelial Cells) and PBMC (Human peripheral blood mononuclear cells) were treated according to the same experimental conditions. As shown in the lower panel of Figure 2A, citrus nanovesicles did not affect the growth of normal cells. In addition, we found that the effects observed on cancer cell proliferation depended on nanovesicles integrity and stability, as their destruction, by boiling or sonication leads to the disappearance of the anti-proliferative effects (Supplementary Figure 1B).

To better evaluate the ability of *Citrus* nanovesicles to inhibit *in vitro* tumor growth, we performed a colony formation assay in methylcellulose. As shown in Figure 2B, A549, SW480 and LAMA84 cells treated with different concentration of nanovesicles formed a lower number of colonies when compared to untreated control cells.

The results reported herein showed that *Citrus* nanovesicles were active against the tumor cell lines A549, SW480 and LAMA84, while they did not affect the proliferation of normal cells, thus confirming the specificity of their effect towards cancer cells.

### *Citrus* nanovesicles activate the expression of pro-apoptotic molecules

**Figure 3:**
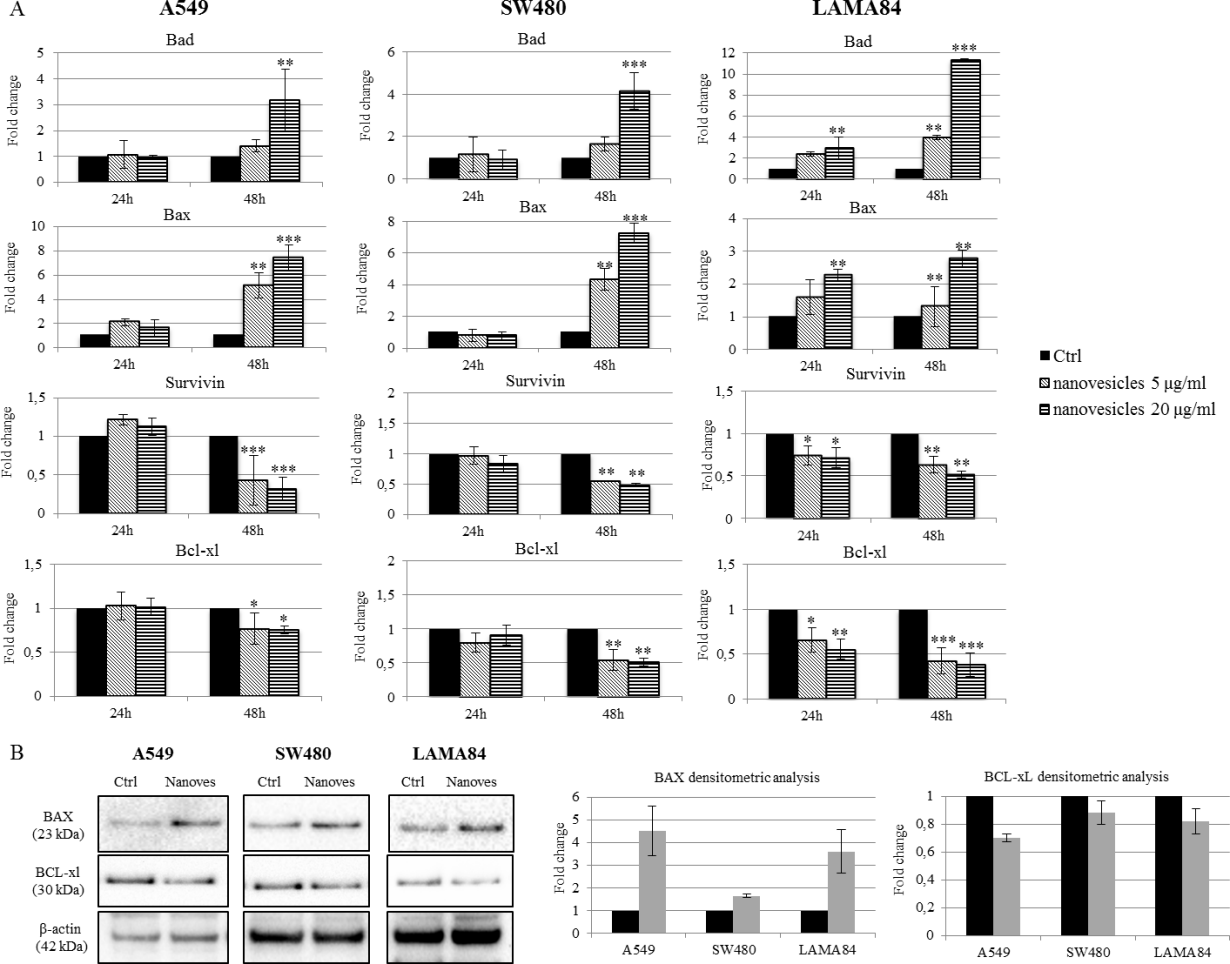
*Citrus* nanovesicles affect the balance between pro- and anti-apoptotic molecules **A.** Real-time PCR analysis was performed on A549, SW480 and LAMA84 cell lines treated for 24 or 48 hours with 5 or 20 μg/ml of nanovesicles to evaluate mRNA levels of the pro-apoptotic and anti-apoptotic genes. The values were plotted as fold change compared to control (untreated cells). Each point represents the mean ± SD of three independent experiments. Asterisks indicate statistically significant values in comparison to control (Ctrl) (*p ≤ 0.05; **p ≤ 0.01; ***p ≤ 0.001). **B.** Western blot analysis was performed on cells treated for 48 h with 20 μg/ml of *Citrus* nanovesicles. Protein levels of the pro-apoptotic (BAX) and anti-apoptotic (BCL-xL) were evaluated. Blots were stripped and subsequently re-probed with an antibody against β-actin to ensure equal loading. Histograms represent densitometry analysis of protein levels in treated cells (Nanoves) versus untreated cells (Ctrl). Each point represents the mean ± SD of three independent experiments.

To evaluate the mechanism by which *Citrus* nanovesicles were able to suppress tumor growth, we tested the expression of different molecules involved in the apoptotic pathway. As shown in Figure 3A, A549, SW480 and LAMA84 cells treated for 24 or 48 hours with lemon nanovesicles showed an increased expression of the pro-apoptotic genes, Bad and Bax, and a reduction of the anti-apoptotic genes Survivin and Bcl-xl, in particular after 48 h of treatment. The increase of BAX protein expression (4.5-fold increase in A549, 1.65-fold in SW480, 3.6-fold in LAMA84) and the decrease of BCL-xL (0.7-fold decrease in A549, 0.88-fold in SW480, 0.82-fold in LAMA84), were also confirmed by western blot analysis (Figure 3B).

### *Citrus* nanovesicles induce TRAIL-mediated cell death

Several studies showed that many natural products exhibited antineoplastic activities related to tumor necrosis factor (TNF)-related apoptosis-inducing ligand (TRAIL) signaling. TRAIL activation has been shown to induce apoptosis in tumor cells while has minimal toxicity against normal tissues [18]. Therefore, to further explore the apoptotic mechanism induced by *Citrus* nanovesicles, we measured the expression of Trail and its receptor Dr5 that tightly control apoptotic processes.

**Figure 4:**
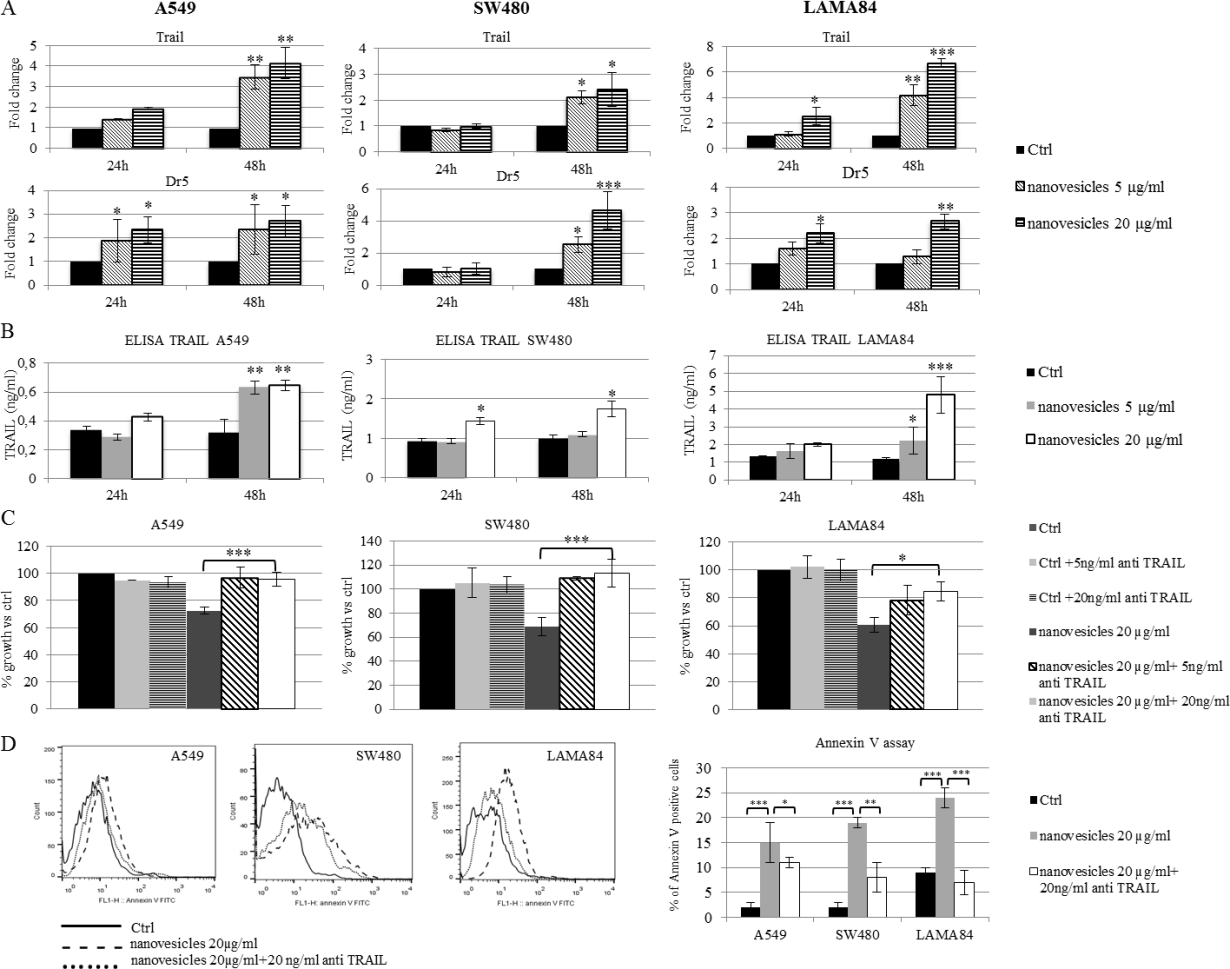
*Citrus* nanovesicles induce TRAIL-mediated cell death **A.** Real-time PCR analysis was performed on A549, SW480 and LAMA84 cell lines treated for 24 or 48 hours with 5 or 20 μg/ml of *Citrus* nanovesicles to evaluate mRNA levels of Trail and Dr5. The values were plotted as fold change compared to control (untreated cells). Each point represents the mean ± SD of three independent experiments. **B.** ELISA was performed to determine TRAIL concentration in the conditioned medium of A549, SW480 and LAMA84 cell lines treated for 24 or 48 hours with 5 or 20 μg/ml of *Citrus* nanovesicles. The values are expressed in ng/ml. Each point represents the mean ± SD of three independent experiments. Asterisks indicate statistically significant values in comparison to control (Ctrl). **C.** Cell growth was measured by MTT assay after 48 h of treatment with 20 μg/ml of nanovesicles in presence or not of 5 or 20 ng/ml of neutralizing anti TRAIL antibodies. The values were plotted as % of growth vs control (untreated cells). Each point represents the mean ± SD of three independent experiments. **D.** Cell death was detected by Annexin V staining after 48 h of treatment with 20 μg/ml of *Citrus* nanovesicles in presence or not of 20 ng/ml of neutralizing anti TRAIL antibodies. Figure shows representative overlay histogram from untreated cells (solid line), cells treated with nanovesicles (dashed line) or with nanovesicles and neutralizing anti TRAIL antibodies (dotted line). Histogram reported % of Annexin V positive cells in samples treated compared to untreated (Ctrl). Asterisks indicate statistically significant differences (*p ≤ 0.05; **p ≤ 0.01; ***p ≤ 0.001).

We found that nanovesicles treatment of cancer cell lines induced an upregulation of Trail (Figure 4A, upper panel) and Dr5 (Figure 4A, lower panel) mRNA levels, with a significant increase after 48 h. As expected, no changes in Trail mRNA expression levels in normal cell line HS5 treated with nanovesicles (Supplementary Figure 1C) were observed. The increased release of TRAIL protein was also confirmed by ELISA assays as shown in Figure 4B. To note, the increase of TRAIL protein level was higher in the CML cell line, LAMA84.

To further demonstrate that the observed nanovesicles-induced cell death was activated through the TRAIL/DR5 pathway, we used human TRAIL neutralizing antibodies. As shown in Figure 4C the co-treatment of cancer cells with *Citrus* juice-derived nanovesicles and TRAIL neutralizing antibody significantly reverted the effects of nanovesicles on tumor cell death. To further investigate if the decrease in cell growth observed after nanovesicles treatment was due to the activation of TRAIL-stimulated apoptotic cell death, Annexin V–FITC fluorescence was measured by flow cytometry on cancer cell lines treated for 48 h with 20 μg/ml of lemon nanovesicles in presence or absence of TRAIL neutralizing antibodies. Figure 4D showed an increase of apoptotic cell death in all the cell lines treated with nanovesicles (up to 15% in A549, 19% in SW480, 24% in LAMA84), while blocking TRAIL led to a reversion of these effects. Overall our data confirmed that lemon-derived nanovesicles stimulated cancer cell death by activating TRAIL-mediated apoptosis.

### *Citrus* nanovesicles reduce the growth of CML xenografts

**Figure 5:**
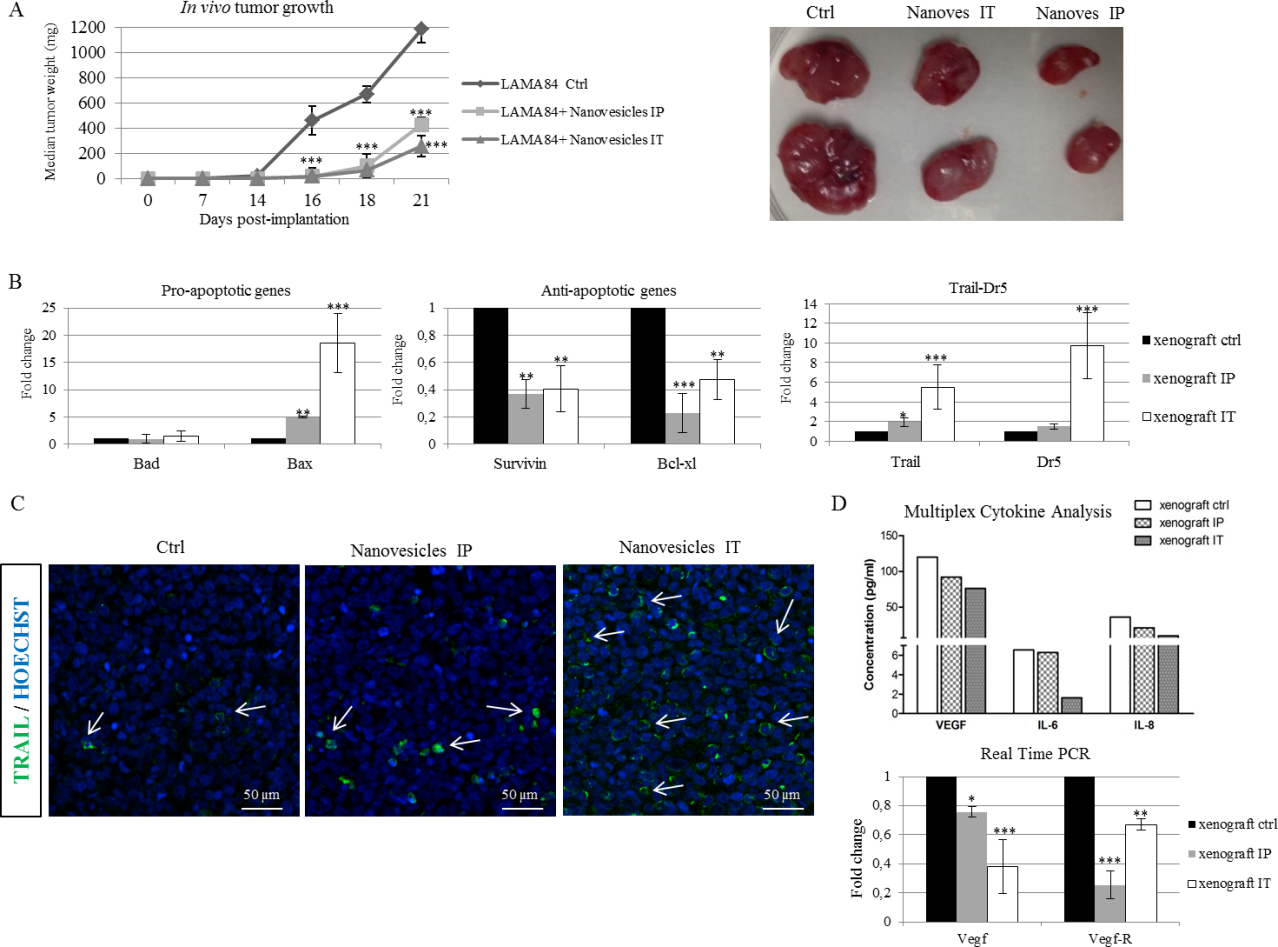
*Citrus* nanovesicles inhibit *in vivo* tumor growth **A.** LAMA84 cells were injected subcutaneously in NOD/SCID mice as described. After palpable tumor formation, mice were treated as described in Materials and Methods. Comparison of the median tumor weight was used as an index of the antitumor efficacy of *Citrus* nanovesicles. Asterisks indicate statistically significant values in comparison to control (Ctrl) (***p ≤ 0.001) **B.** mRNA levels of pro-, anti-apoptotic genes, Trail and Dr5 were evaluated in samples from mice xenografts. The values were plotted as fold change compared to xenograft control. Each point represents the mean ± SD for three independent experiments. Asterisks indicate statistically significant values in comparison to control (Ctrl) (**p ≤ 0.01; ***p ≤ 0.001). **C.** Representative images of confocal fluorescence microscopy show TRAIL (green) immunolabeling in paraffin sections from xenografts. Nuclear counterstaining was performed using Hoescht (blue). Arrows indicate TRAIL positive cells. **D.** Multiplex cytokine evaluation of VEGF-A, IL6 and IL8 in the serum of mice treated or not with nanovesicles. The values are expressed in pg/ml (upper panel). mRNA levels of Vegf-A and Vegf-A receptor were evaluated in samples from mice xenografts (lower panel). The values were plotted as fold change compared to control. Each point represents the mean ± SD for three independent experiments. Asterisks indicate statistically significant values in comparison to control (Ctrl) (*p ≤ 0.05; **p ≤ 0.01; ***p ≤ 0.001).

The ability of *Citrus* nanovesicles to reduce tumor growth was also tested in an *in vivo* tumor xenograft model. LAMA84 cells were inoculated subcutaneously in NOD/SCID mice; one week post cell injection, mice were treated locally (intra tumor, IT) or intraperitoneally (IP) three times a week with vehicle (PBS) or lemon-derived nanovesicles. At the end of treatment regime, mice were sacrificed and the tumors removed. Strikingly, in Figure 5A we showed that tumor growth was reduced in mice treated with *Citrus* nanovesicles, both locally and intraperitoneally (left panel), leading to the formation of smaller tumors compared with control mice (right panel). There are no statistically significant differences between the two different groups of mice treated with nanovesicles.

In order to confirm our *in vitro* data, we tested whether *in vivo* tumor size reduction was stimulated by TRAIL activation. Real-time PCR analysis of the mRNAs isolated from *in vivo* xenograft tumors showed an increase of Trail and Dr5 mRNA. In addition, we found that nanovesicle administration led to an increase of the pro-apoptotic gene Bax in nanovesicle-treated mice concomitantly to a decrease of the anti-apoptotic genes Survivin and Bcl-xl (Figure 5B); to note, the differences in the expression of Trail and its receptor were strongest in xenografts from mice treated locally. Furthermore, our results were confirmed by immunofluorescence analysis of TRAIL; we found an increased number of TRAIL positive cells in the tumors of mice treated locally or intraperitoneally with nanovesicles when compared with control mice (Figure 5C).

To further investigate other mechanisms that might be involved in nanovesicle-dependent tumor inhibition, we performed a multiplex cytokine analysis and we found that the administration of nanovesicles led to the decrease of the pro-angiogenic factors, such as VEGF-A, IL6 and IL8 in the serum of treated-mice compared with untreated ones (Figure 5D, upper panel). To note, the reduction of these factors is more significant in locally treated mice (IT). The downregulation of VEGF-A, together with the decrease of its receptor, was also validated by Real time PCR in the xenograft tumors, as shown in Figure 5D, lower panel. Interestingly, we found a comparable decrease of the same pro-angiogenic factors in the conditioned medium of LAMA84 cell line treated with nanovesicles (Supplementary Figure 2).

All together, these data suggested that *Citrus limon* nanovesicles are able to reduce *in vivo* tumor growth by TRAIL-mediated apoptosis and by inhibition of the secretion of cytokines involved in angiogenesis.

### *In vivo* distribution of *Citrus* nanovesicles

**Figure 6:**
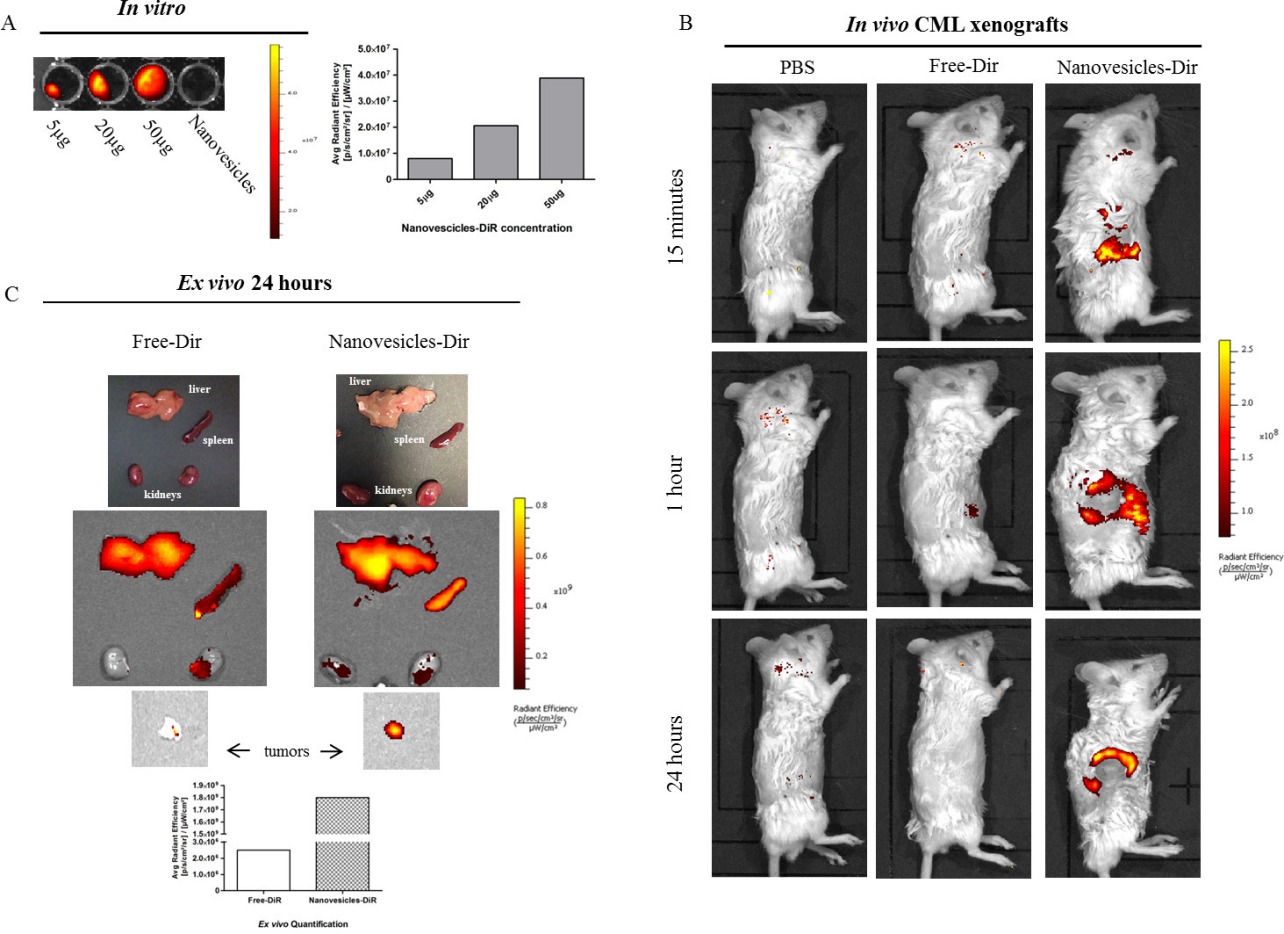
*In vivo Citrus* nanovesicles biodistribution **A.** Representative *in vitro* fluorescence images of DiR-labeled *Citrus* nanovesicles dilutions from 50 to 5 μg of nanovesicles in 150 μl of PBS; the quantification of fluorescence signal was calculated in the entire area of each well through the use of ROIs. Data are expressed as average radiance efficiency ([p/s/cm^2^/steradian]/[μW/cm2]). **B.** NOD/SCID mice bearing CML xenograft tumors in the right flank were injected intraperitoneally with PBS, Free-DiR, 50 μg Nanovesicles-DiR in a volume of 150 μl PBS. Mice were imaged at 15 min, 1 h and 24 h post injection. A scale of the radiance efficiency is presented to the right of each live mouse image. **C.** Organs and tumors were excised and imaged after 24 h. A scale of the radiance efficiency is presented to the right. Histogram represents *ex vivo* quantification of tumor fluorescence.

In order to test *Citrus* nanovesicle *in vivo* distribution, nanoparticles were labeled with the lipophilic fluorescent tracer DiR (1, 10-dioctadecyl-3, 3, 30, 30-tetramethylindotricarbocyanine). As reported in Figure 6A, Nanovesicles-DiR, ranging from 5 to 50 μg of vesicles, showed a fluorescence signal that correlated linearly with nanovesicles concentration.

To assess if nanovesicles inhibited tumor growth by reaching tumor site, NOD/SCID mice were treated intraperitoneally with *Citrus* nanovesicles labeled with the lipophilic fluorescent tracer DiR, with Free-DiR, or with PBS, one week after subcutaneously injection of CML cells. Mice were imaged using an IVIS Optical Imaging System at 15 minutes, 1 and 24 h post injection. At the 24-hour time point, mice were sacrificed and organs excised. As shown in Figure 6B and Supplementary Figure 3A, labeled nanovesicles (Nanovesicles-DiR) quickly reached tumor tissue and accumulated starting at 15 minutes, 1 h and up to 24 hour time point, while the Free-DiR never reached the tumor site.

Analysis of organs excised 24h post injection showed that both Free-DiR and Nanovesicles-Dir are significantly taken up by liver, spleen and partially by kidneys (Figure 6C and Supplementary Figure 3B). A similar distribution of both Free-DiR and Nanovesicles-Dir were observed in organs excised from healthy mice (Supplementary Figure 3C). Analysis of tumors (from mice injected with Free-DiR or with Nanovesicles-DiR) indicates that nanovesicles are internalized by tumors and remain in the tumor mass, while no signal accumulation was observed in the tumors from mice treated with the probe alone (Free-DiR) (Figure 6C).

Taken together, these results clearly suggest the specificity of nanovesicles to reach tumor sites *in vivo*.

## DISCUSSION

Currently, many natural compounds emerged as alternative strategies for cancer prevention and therapy [19]. Natural compounds are used in monotherapy or in association with chemotherapeutic drugs, thus allowing the use of lower dosage of chemotherapeutic agent in order to overcome drug resistance and toxicity on normal tissues.

Numerous data in literature showed that *Citrus* fruit, commonly used in traditional medicine in China and other countries, has an anti-proliferative effect in many types of cancers [20, 21]; however, it is still unknown how this occurs and through which molecular mechanisms.

Evidences support the occurrence of multivesicular bodies-mediated secretion of exosome-like vesicles in plants [6], but the understanding of their functional role in cross-kingdom interaction requires further exploration. Zhang and colleagues have reported that grape nanoparticles are taken up by mouse intestinal macrophages, thus inducing the expression of anti-oxidant genes and suppressing the production of pro-inflammatory cytokines [7]. However, the role of plant-nanovesicles to influence cancer progression has never been described.

Our findings showed that we were able to isolate a homogeneous population of nanovesicles from *Citrus limon*-juice, with dimension, morphology and protein contents attributable to exosome-like nanoparticles [17]. Furthermore, we showed that these nanovesicles are stable and with a functional role in cross-kingdom communication. In particular, for the first time, we showed that they inhibit cancer cell growth without affecting normal cells. The mechanism of action of many anticancer compounds is based on their ability to induce apoptosis. Many natural products exert their apoptotic effects by inducing TRAIL-mediated cell death [22, 23]. It has been largely described that TRAIL selectively induces apoptosis of cancer cells without affecting normal cells [16, 24, 25]. Furthermore, it has been described that many types of tumor are TRAIL-resistant due to the low expression levels of TRAIL receptors [26]. Therefore, the development of agents that can sensitize cells to TRAIL-mediated cell death are needed. Here we showed that the treatment of lung, colon and leukemia cancer cells with *Citrus* nanovesicles affects pro-and anti-apoptotic pathways, leading to the increase in the mRNA levels of the pro-apoptotic molecules Bad and Bax, together with the decrease of pro-survival molecules, such as Survivin and Bcl-xl. Furthermore, we observed an increased expression of TRAIL-receptor, Dr5, in cancer cell lines treated with nanovesicles, together with the increase and release of TRAIL, thus hypothesizing an autocrine loop induced by lemon vesicles that leads to cancer cell death.

To validate our *in vitro* findings showing the pro-apoptotic effects of nanovesicles on tumor cell lines, we used an *in vivo* xenograft model of chronic myeloid leukemia (CML). Data reported here showed that the administration of *Citrus* nanovesicles strongly suppressed tumor growth and we confirmed, *in vivo*, that this effect was due not only to TRAIL-mediated apoptosis but also to the inhibition of angiogenic processes, as shown by the reduced levels of pro-angiogenic cytokines VEGF-A, IL6 and IL8. Interestingly, our data are consistent with our previous results demonstrating the ability of exosomes-induced IL8 to stimulate CML cell proliferation and survival [27]. Moreover, through *in vivo* optical imaging analysis we proved that nanovesicles exert these effects by reaching tumor site. Overall, our data showed that the pro-apoptotic effects of *Citrus limon* may be attributed to structural components and in particular to exosomes-like nanovesicles. Furthermore, the possible mechanism by which nanovesicles exert the *in vitro* and *in vivo* antineoplastic activity involves TRAIL-mediated pathways as well as angiogenic inhibition.

In summary, we have identified for the first time nanovesicles from *Citrus limon* juice with antineoplastic potential. Our findings open to the possibility to develop new anticancer strategies based on the use of plant-edible nanovesicles.

## MATERIALS AND METHODS

### Ethics statement

All animal experiments were conducted in full compliance with University of Palermo and Italian Legislation for Animal Care. The Dipartimento di Biopatologia e Biotecnologie Mediche (DiBiMed) Review Board approved this study.

### Cell culture and reagents

The human chronic myeloid leukemia cell line, LAMA84, was obtained by DSMZ (Braunschweig, Germany). The human colorectal adenocarcinoma cell line, SW480, the human lung carcinoma cell line, A549, and the human bone marrow-derived stromal cell line, HS5, were obtained by ATCC (Manassas, VA, USA). LAMA84, SW480 and A549 cell lines were cultured in RPMI 1640 medium (Euroclone, UK), HS5 (Human bone marrow stromal cells) cell line was cultured in DMEM high glucose (Euroclone, UK). Human Umbilical Vein Endothelial Cells (HUVEC) were obtained from Lonza and grown in Endothelial Growth Medium (EGM, Clonetics, Verviers, Belgium). Human peripheral blood mononuclear cells (PBMC) were isolated using Ficoll Paque (GE Helthcare Bio Science, Uppsala, Sweden). Anti-TRAIL neutralizing antibody was from AbCam (Cambridge, UK). All other reagents were purchased from Sigma-Aldrich (St. Louis, MO, USA), if not cited otherwise.

### Nanovesicles preparation

Nanovesicles were isolated from *Citrus limon* L. juice. Fruits, obtained from a private farmer, were carefully washed in water and manually squeezed. The juice was sequentially centrifuged at 3,000 × g for 30 minutes, and 10,000 × g for 1 hour. The supernatant was filtered at 0.8 μm and 0.45 μm pore filter and centrifuged at 16,500 × g for 3 hours. The supernatant was then centrifuged at 120,000 × g for 90 minutes in a Type 70 Ti, fixed angle rotor, the pellet was suspended in 1 ml PBS and transferred to a 30% sucrose/D2O cushion. Vesicles contained in the cushion were recovered, washed several times, ultracentrifuged for 90 min in PBS and collected for use. Nanovesicles quantification was determined with the Bradford assay (Pierce, Rockford, IL, USA). On average, we recovered 600 micrograms of vesicles from 240 ml of *Citrus* juice.

### Dinamic light scatter (DLS)

Nanovesicles size distribution was determined by dynamic light scattering (DLS) experiments. Collected nanovesicle samples were diluted to avoid inter-particle interaction and placed at 20°C in a thermostatic cell compartment of a Brookhaven Instruments BI200-SM goniometer, equipped with a solid-state laser tuned at 532 nm. Scattered intensity autocorrelation functions were measured by using a Brookhaven BI-9000 correlator and analyzed in order to determine the size distribution [28]. The size at the maximum of the distribution (moda) is reported as a significant average size.

### Transmission electron microscopy (TEM)

Nanovesicles resuspended in PBS (10μg) were spotted onto carbon-coated grids, fixed in 1% glutaraldehyde and stained in 2% phosphotungstic acid. The preparation obtained was examined immediately using a JEOL JEM-1400 Plus transmission electron microscope, at 110 kV.

### Sample preparation for proteomic analysis

Gel-free and gel-based protein fractionation procedures were employed and combined with LC-MS/MS analysis in order to increase the proteome coverage of *Citrus limon* nanovesicles.

In gel-free approach nanoparticles isolated from *Citrus limon* juice were processed using 50% 2,2,2-trifluoroethanol (TFE) in PBS and incubated with constant shaking for 1 h at 60°C. Reduction was performed with 5 mM dithiothreitol (DTT) for 30′ at 60°C and alkylation with 25 mM iodoacetamide (IAA) for 30′ in the dark at room temperature. Prior to trypsin addition, sample was diluted with four-volumes of 100 mM NH4HCO3 pH 8.0. Proteins were digested overnight at 37°C using sequencing-grade modified porcine trypsin (Pierce). After overnight incubation, digestion was stopped by adding 20 ul of 90% FA and peptide mixture centrifuged at 14000g for 10′ at 4°C.

In the gel-based approach (GeLC-MS/MS), nanovesicle proteins extracted as previously described [29] were separated by 10% SDS-PAGE and stained with colloidal Coomassie Blue solution. Gel lane was cut into eight gel slices of similar size and further cut into about 1 mm3 particles; in-gel digestion protocol was adapted from Shevcenko *et al*. [30]. Peptides obtained from the two procedures were finally dried down with a speed vacuum centrifuge and desalted by solid phase extraction using C18 Macrospin Columns.

### Liquid chromatography-tandem mass spectrometry

The MS analysis was performed using a Triple TOF 5600 Plus System (AB Sciex, Framingham, U.S.A.) equipped with an Eksigent Nanoflow binary gradient HPLC system and a nanospray III ion source. Two microliters of sample were injected on a reversed-phase trap column for peptide cleanup and pre-concentration, employing a mobile phase, from loading pump, containing 0.1% v/v FA in water at a flow rate of 5 ul/min. Peptides were then eluted onto the C18 analytical column, equilibrated at 40°C with a solvent A (0, 1% FA in water) at a flow rate of 250 nL/min, and separated using a gradient method according to which solvent B (0, 1% FA in acetonitrile) was linearly increased from 10% to 28% within 60 min and then to 60% within 15 min; afterwards, phase B was further increased to 95% within 1 min. Then, phase B was maintained at 95% for 5 min to rinse the column. Finally, B was lowered to 10% over 1 min and the column reequilibrated for 18 min (100 min total run time).

The eluting peptides were on-line sprayed in the Triple TOF 5600 Plus mass spectrometer, that it is controlled by Analysts 1.6.1 software (AB SCIEX, Toronto, Canada).

Data were acquired using an ion spray voltage of 2.7 kV, curtain gas set at 35, GS1 1 and GS2 0 PSI nitrogen flow, source temperature 80°C. MS/MS spectra were collected using Information Dependent Acquisition (IDA); precursor ions were selected across the mass range of 350 to 1250 m/z in high resolution mode (>30, 000) using 250 ms accumulation time per spectrum. A maximum of 35 precursors per cycle from each MS spectrum, with charge state from 2 to 5, were selected for fragmentation, if exceeding a threshold of 70 counts per second (cps), with 100 ms minimum accumulation time for each precursor and dynamic exclusion for 15 s. Tandem mass spectra were recorded in high sensitivity mode (resolution > 15,000) with rolling collision energy.

### Protein identification and data analysis

Raw MS/MS data files from Analysts 1.6 software were submitted to ProteinPilot™ 4.5 software (AB SCIEX, Toronto, Canada), using the Paragon Algorithm and Uniprot′s Citrus database (39096 entries, July 2014); For all analyses, the search was performed with the following settings: (1) Sample Type: identification; (2) Cysteine Alkylation: Iodocetamide; (3) Digestion: Trypsin; (4) Instrument: TripleTOF 5600; (5) Special factors: /Gel-based ID (for samples from GeLC-MS/MS analysis) /None (for samples from LC-MS/MS); (6) Species: None; (7) Search Effort: Thorough ID; (8) FDR Analysis: Yes. Moreover, further analysis were performed with the KEGG Orthology (KO) system (http://www.genome.jp/kegg/ko.html) and the exosome database ExoCarta (http://exocarta.org) [31].

### Uptake of *Citrus* nanovesicles by A549 and LAMA84 cells

*Citrus* nanovesicles were isolated as described above and labeled with PKH26 (Sigma-Aldrich, St. Louis, MO, USA) for 10 min at room temperature. Labeled nanovesicles were washed twice in PBS and resuspended in complete medium. A549 and LAMA84 cells were grown on coverslips coated with type I collagen (Calbiochem, Darmstadt, Germany) and treated with 20 μg/ml of labeled vesicles for 3 or 6 hours at 4°C or 37°C. Cells were stained with Actin Green 488 (Molecular probes, Life Technologies, California, USA). Nuclei were stained with Hoechst 3342 (Molecular probes, Life Technologies) and analysed by confocal microscopy.

### Viability assay (MTT assay)

Cell viability was assessed with Methyl-thiazol-tetrazolium (MTT) assay as previously described [32]. Briefly, cells were seeded at a density of 0.1 × 10^6^ in a 96-well plate and exposed to escalating doses of *Citrus* nanovescicles (5–20 μg/ml) for 24, 48 or 72 hours, and in the presence or not of 5, 20 μg/ml of neutralizing TRAIL ab. MTT assay was also performed on A549 cell line treated for 24, 48 or 72 hours with 5 or 20 μg/ml of boiled or sonicated nanovesicles. The absorbance was measured at 540 nm. Means and standard deviations generated from three independent experiments are reported as the percentage of growth versus control (untreated cells). Cell proliferation curves were derived from these data with Microsoft Excel software.

### Colony formation assay

A549, SW480 and LAMA84 cells were plated in 6-well (2000 cells/ml/well) in Iscove′s-methylcellulose medium (Methocult H4230, Stem Cell Technologies, Vancouver, Canada) containing or not nanovesicles (5, 20 μg/ml). After 14 days of culture, colonies were observed by phase-contrast microscopy and photographed. The area of twenty colonies per condition was measured with the IMAGE-J software (http://rsbweb.nih.gov/ij/).

### Annexin V assay

To detect tumor cell apoptosis, an Annexin V-fluorescein isothiocyanate (FITC) assay was used. Specifically, LAMA84, SW480 and A549 cells were seeded into 6-well plates, cultured for 48 h and treated with 20 μg/ml of *Citrus* vesicles in the presence or not of neutralizing TRAIL ab (20 ng/ml). After incubation, cells were washed in PBS twice and apoptosis assays were performed as follows. Cells were resuspended in cold 1X Annexin V binding buffer (10x: 0.1 M Hepes pH 7.4; 1.4 M NaCl; 25 mM CaCl2), transferred in a FACS tube and mixed with 5 μl of Annexin V-FITC (BD-Biosciences, San Jose, CA). The cells were then incubated at room temperature in the dark for 15 min. After adding 400 μl of annexin V binding buffer, the samples were subjected to flow cytometry analysis to detect cell apoptosis levels. Cells positive for Annexin V-FITC, were considered to represent apoptotic cells. Stained cells were acquired on FACS Calibur (BD Biosciences San Jose, CA) and analysed using FlowJo software (Tree Star, Ashland OR).

### RNA extraction and real-time PCR

LAMA84, A549 and SW480 cells were grown in 12-well plates and treated with 5 or 20 μg/ml of *Citrus* vesicles for 24 or 48 hours. Tumor biopsies soon after removal were stored in RNAlater solution (Applied Biosystems, Foster City, California, USA). Each sample was lysed in a tissue homogenizer. RNA was extracted using the commercially available Illustra RNAspin Mini Isolation Kit (GE Healthcare, Little Chalfont, Buckinghamshire, UK), according to manufacturer′s instructions. Total RNA was reverse-transcribed to cDNA using the High Capacity cDNA Reverse Transcription Kit (Applied Biosystem). RT-QPCR was performed in 48-well plates using the Step-One Real-Time PCR system (Applied Biosystem). For quantitative SYBR^®^Green realtime PCR, the following primers were used:

GAPDH (5′ATGGGGAAGGTGAAGGTCG3′, 5′G GGTCATTGATGGCAACAATAT3′),

Bad (5′CCGAGGAGCAGGAAGACTC′3, 5′GGT AGGAGCTGTGGCGACT′3),

Bax (5′CCTGTGCACCAAGGTGCCGGAACT3′, 5′CCACCCTGGTCTTGGATCCAGCCC3′),

Survivin (5′CTCAAGGACCACCGCATCTC′3, 5′C AGCCTTCCAGCTCCTTGAA′3),

Bcl-xl (5′CTGAATCGGAGATGGAGACC′3, 5′TG GGATGTCAGGTCACTGAA′3),

Trail (5′GCTCTGGGCCGCAAAAT′3, 5′ TGCAAG TTGCTCAGGAATGAA′3),

Dr5 (5′ GGGCCACAGGGACACCTT ′3, 5′ GCATC TCGCCCGGTTTT′3),

Vegf-A (5′ CGAGGGCCTGGAGTGTGT′3, 5′CGC ATAATCTGCATGGTGATG ‘3)

Vegf-A Receptor (5′ CGGTCAACAAAGTCGGGA GA ′3, 5′CAGTGCACCACAAAGACACG ‘3),

all obtained from Invitrogen (Foster City, CA, USA). Real-time PCR was performed in triplicates for each data point. Relative changes in gene expression between control and treated samples were determined with the ΔΔCt method. Levels of the target transcript were normalized to a GAPDH endogenous control, constantly expressed in all samples (ΔCt). For ΔΔCt values, additional subtractions were made between treated samples and control ΔCt values. Final values were expressed as fold of induction.

### Western blot

A549, SW480, LAMA84 cells were treated for 48 h with 20 μg/ml of *Citrus* nanovesicles. Total protein cell lysates were obtained and analyzed by SDS-PAGE followed by Western blotting. Antibodies used in the experiments were anti-BAX, BCL-xL and β-actin (all from Santa Cruz Biotechnology, CA, USA).

### ELISA

A549, SW480, LAMA84 cells conditioned medium was collected from cells treated with 5, 20 μg/ml of *Citrus* nanovesicles for 24 or 48 h. Conditioned medium aliquots were centrifuged to remove cellular debris and then TRAIL protein concentrations were quantified using the ELISA kit (Uscn life Science Inc., Houston, TX, USA), according to manufacturer′s protocol.

### CML mouse xenograft

Male NOD/SCID mice, four-to-five weeks old, were purchased from Charles River (Charles River Laboratories International, Inc, MA, USA) and acclimated for a week prior to experimentation. Mice received filtered water and sterilized diet *ad libitum*. Animals were observed daily and clinical signs were noted. Each mouse was inoculated subcutaneously in the right flank with viable single human LAMA84 cells (2 × 10^7^) suspended in 0.2 ml of PBS. The day of injection was considered as Day 0. On Day 7, when tumors were palpable, mice were randomly assigned to three groups of four and were treated with *Citrus* nanovesicles (50 μg/mouse, three days a week for two weeks) administered intraperitoneally (IP) or in the intratumor (IT) site or with vehicle (PBS). Tumor xenografts were measured and the mice were weighed three times a week starting on Day 7. Tumor volume was determined by caliper by using the following formula: L × W^2^/2 = mm^3^ where L and W are the longest and shortest perpendicular measurements in millimeters, respectively. The same formula was used to calculate tumor weights assuming that 1 mm^3^ = 1 mg. Animals were euthanized at the end of treatment, the tumor removed, and the tumor weights measured. Blood was collected by post-mortem cardiac puncture, centrifuged at 1500 × g for 15 min and the supernatant (serum) was collected and stored at −20°C. Xenografts were resuspended in RNA later for further RNA isolation or in 10% formalin for immunohistochemical analysis.

### Immunofluorescence

Mice xenografts were harvested and immediately fixed with 10% formalin. 5-μm-thick paraffin-embedded tumor sections were used for Immunofluorescence. Briefly, slides were rehydrated and subsequently retrieved for 15 minutes at 95°C in NaCitrate 10 mM pH 6. After antigen retrieval, tumor sections were permeabilized with 0.2% TritonX-100 in PBS for 10 minutes at room temperature and then blocked with Dako protein block serum free (Dako). Sections were incubated with mouse monoclonal antibody anti-TRAIL (Abcam, catalog # ab 10516) diluted 1:50 in blocking solution over night at 4°C. After washes in 0.2% TritonX-100 in PBS, sections were incubated with Alexa-fluor 488 donkey anti-mouse (Life technologies) diluted 1:500 in blocking solution for 1 hour at room temperature in the dark. Slides were subsequently counterstained with Hoechst 3342 and mounted for confocal analysis.

### Multiplex cytokine analysis

Levels of VEGF-A, IL-6 and IL8 were determined in mice serum samples and in the conditioned medium of LAMA84 cell line using the Bio-Plex Multiple Cytokine Assay (Bio-Rad, Hercules, CA), according to the manufacturer′s instructions. Briefly 50 μL of samples and scalar concentrations of the assay standards were added in duplicates to a 96-well plate containing magnetic beads. The plate was incubated for 1 hour followed by washing steps; the plate was subsequently coated with biotinylated detection antibody solution and incubated for 30 minutes. After the 30 minutes incubation, the plate was washed and streptavidin-conjugated phycoerythrin was added to the 96-well plates and incubated for 10 minutes. The plate was washed after this final incubation step and assay buffer was added to each well. Data was acquired using the Bio-Plex® 200 Systems. A standard curve was derived using the different concentrations of the assay standards. Data were analyzed using Bio-Plex Manager Software.

### *In vivo* distribution of *Citrus* nanovesicles

Nanovesicle labeling procedure: DiR was used to fluorescently label the lipid bilayer of *Citrus* nanovesicles. Briefly, nanovesicles were directly labeled with 1 μM Vybrant Cell Tracers DiR (Life Technologies) then washed in PBS. To test the efficiency of DiR conjugation, 5, 10 and 50 μg of labeled nanovesicles were *in vitro* evaluated by using a cooled charge-coupled device (CCD) camera imaging (IVIS Lumina; PerkinElmer LifeSciences) using the appropriate filter (exc = 710 nm; em = 780 nm). The same region of interest (ROI) was applied in the entire area of each well on all the fluorescent signal. Data were expressed as Average Radiant Efficiency [p/s/cm^2^/sr] / [μW/cm^2^].

*In vivo* imaging: healthy mice or mice bearing CML xenografts were monitored daily. Prior to nanovesicle injection and optical imaging acquisitions, the ventral hair of mice was removed. For *in vivo* imaging studies, different groups of animals were analyzed: healthy mice or mice bearing tumors injected with a) 150 μl PBS; b) Free Dir probe (1 μM in 150 μl PBS); c) 50 μg of Dir-labeled nanovesicles in 150 μl PBS.

Briefly, for the detection of fluorescence, mice were anesthetized and then injected IP with PBS, Free DiR or DiR labeled-nanovesicles. Mice images were acquired after 15 minutes, 1 h or 24 h from injection. Following fluorescent background subtraction, images were analyzed and scaled after completion of all acquisitions, using appropriate computer software (Living Image Software; PerkinElmer LifeSciences). All mice were imaged with identical instrument settings. Following the last acquisition, the animals were sacrificed and the organs (spleen, liver, kidneys and tumor) were collected and acquired with same imaging system. The scale bar was expressed as Average Radiant Efficiency [p/s/cm^2^/sr] / [μW/cm^2^] which is a calibrated measurement of photon emission.

### Statistical analysis

Data are expressed as means ± SD of three independent experiments. Statistical analysis was done with a paired sample *t*-test. Differences were considered significant when *p* ≤ 0.05.

## SUPPLEMENTARY FIGURES AND TABLE




