# Supplementary material for: Citrus limon-derived nanovesicles inhibit cancer cell proliferation and suppress CML xenograft growth by inducing TRAIL-mediated cell death
Source: Oncotarget. 2015 May 18;6(23):19514–27. doi: 10.18632/oncotarget.4004 (PMC4637302; doi:10.18632/oncotarget.4004)
Supplement: Supplementary file 1 [file oncotarget-06-19514-s001.pdf]

SUPPLEMENTARY FIGURES AND TABLE

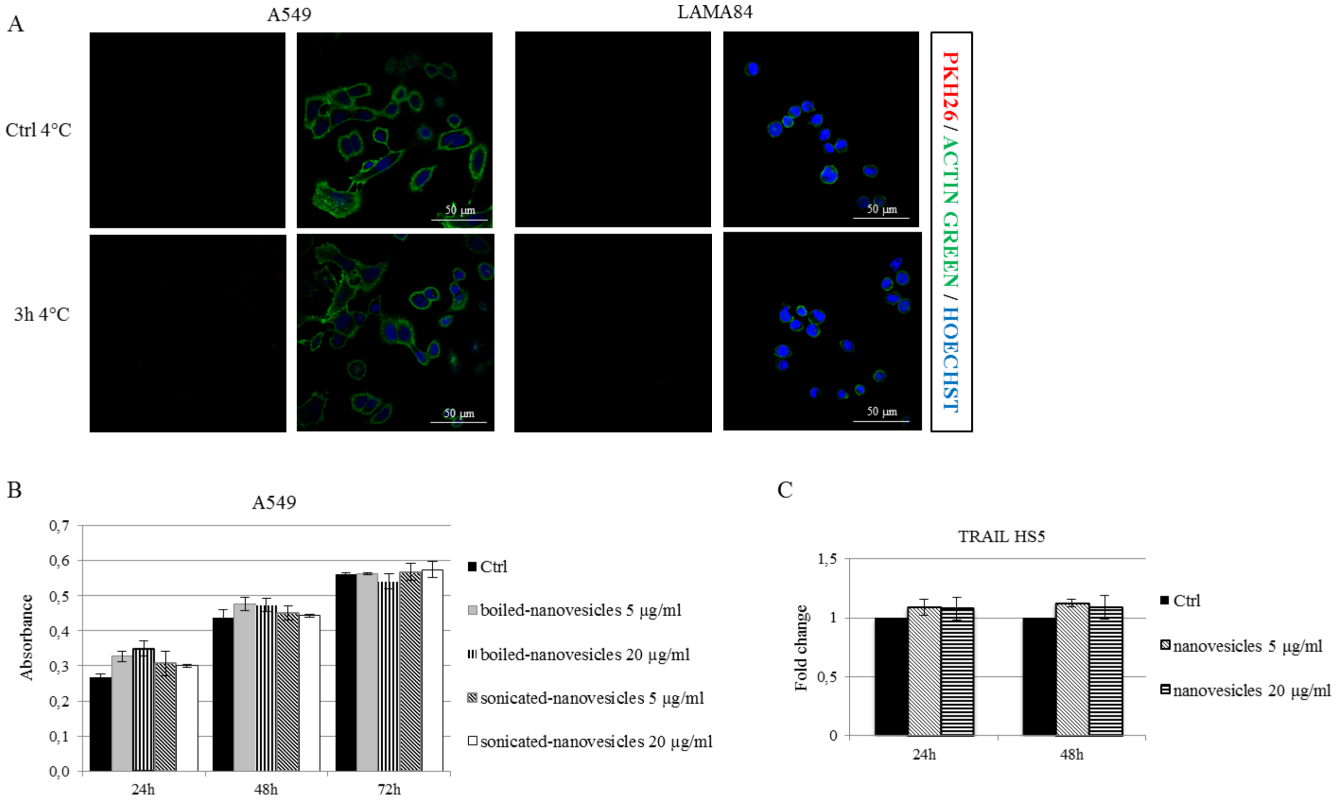

Supplementary Figure 1: A. Analysis at confocal microscopy of A549 (left panel) or LAMA84 cells (right panel) treated, for 3 hours, with 20 µg/ml of Citrus nanovesicles at 4°C, compared with untreated cells (Ctrl). Cells were stained with Actin Green 488 (green), nuclear counterstaining was performed using Hoechst (blue), nanovesicles were labeled with PKH26 (red). **B.** MTT assay after 24, 48, 72 h of treatment with 5 or 20 µg/ml of boiled-nanovesicles or sonicated-nanovesicles. The values were plotted as absorbance. Each point represents the mean ± SD of three independent experiments. **C.** Real-time PCR analysis was performed on HS5 cell lines treated for 24 or 48 hours with 5 or 20 µg/ml of Citrus nanovesicles to evaluate mRNA levels of Trail. The values were plotted as fold change compared to control (untreated cells). Each point represents the mean ± SD of three independent experiments.

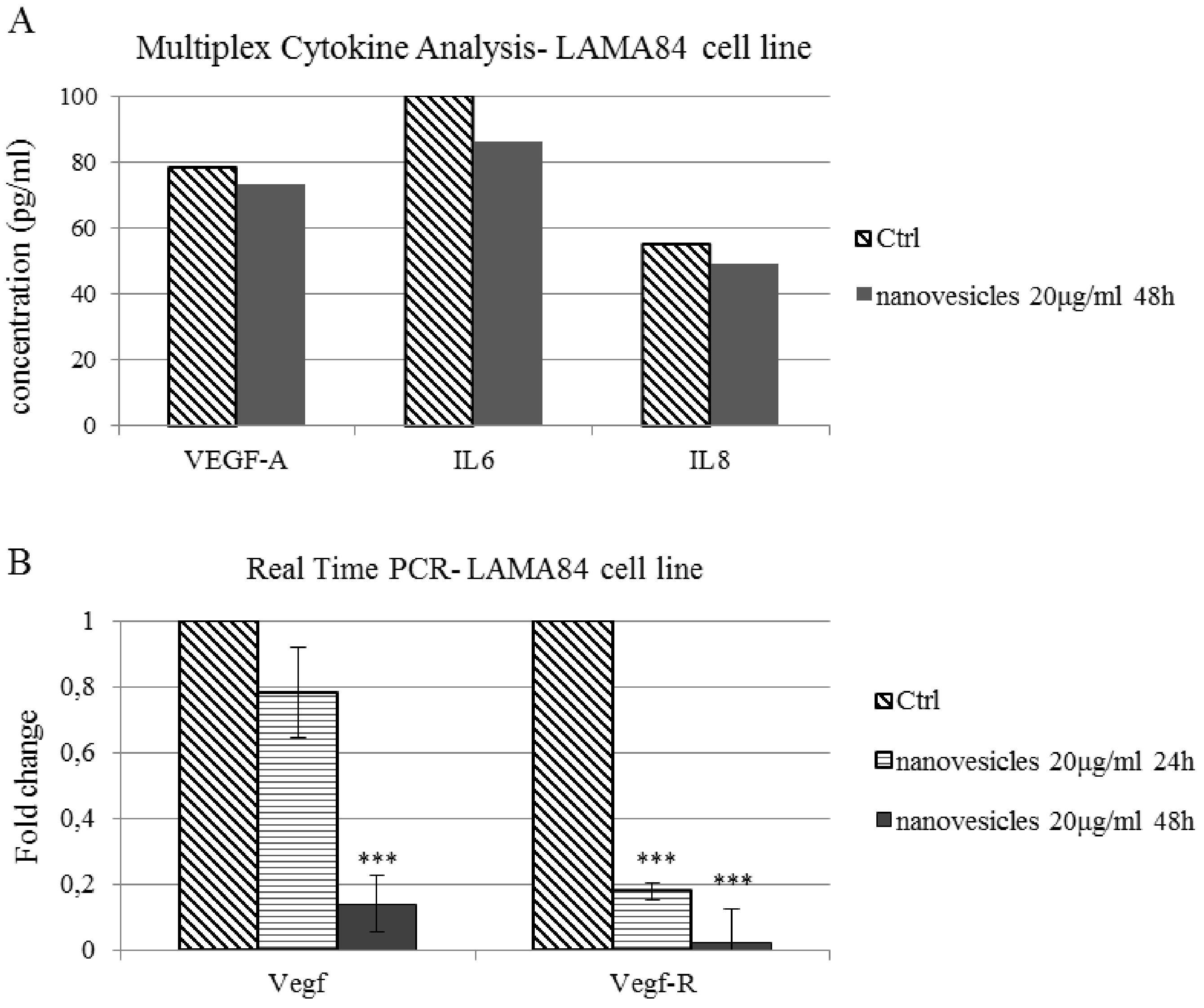

Supplementary Figure 2: A. Multiplex cytokine evaluation of VEGF-A, IL6 and IL8 in the conditioned medium of LAMA84 cell line treated or not with 20 µg/ml of Citrus nanovesicles for 48 h. The values are expressed in pg/ml. **B.** mRNA levels of Vegf-A and Vegf-A receptor evaluated in LAMA84 cell line treated with 20 µg/ml of Citrus nanovesicles for 24 or 48 h. The values were plotted as fold change compared to control (untreated cells). Each point represents the mean ± SD for three independent experiments. Asterisks indicate statistically significant values in comparison to control (Ctrl) (***$p \leq 0.001$).

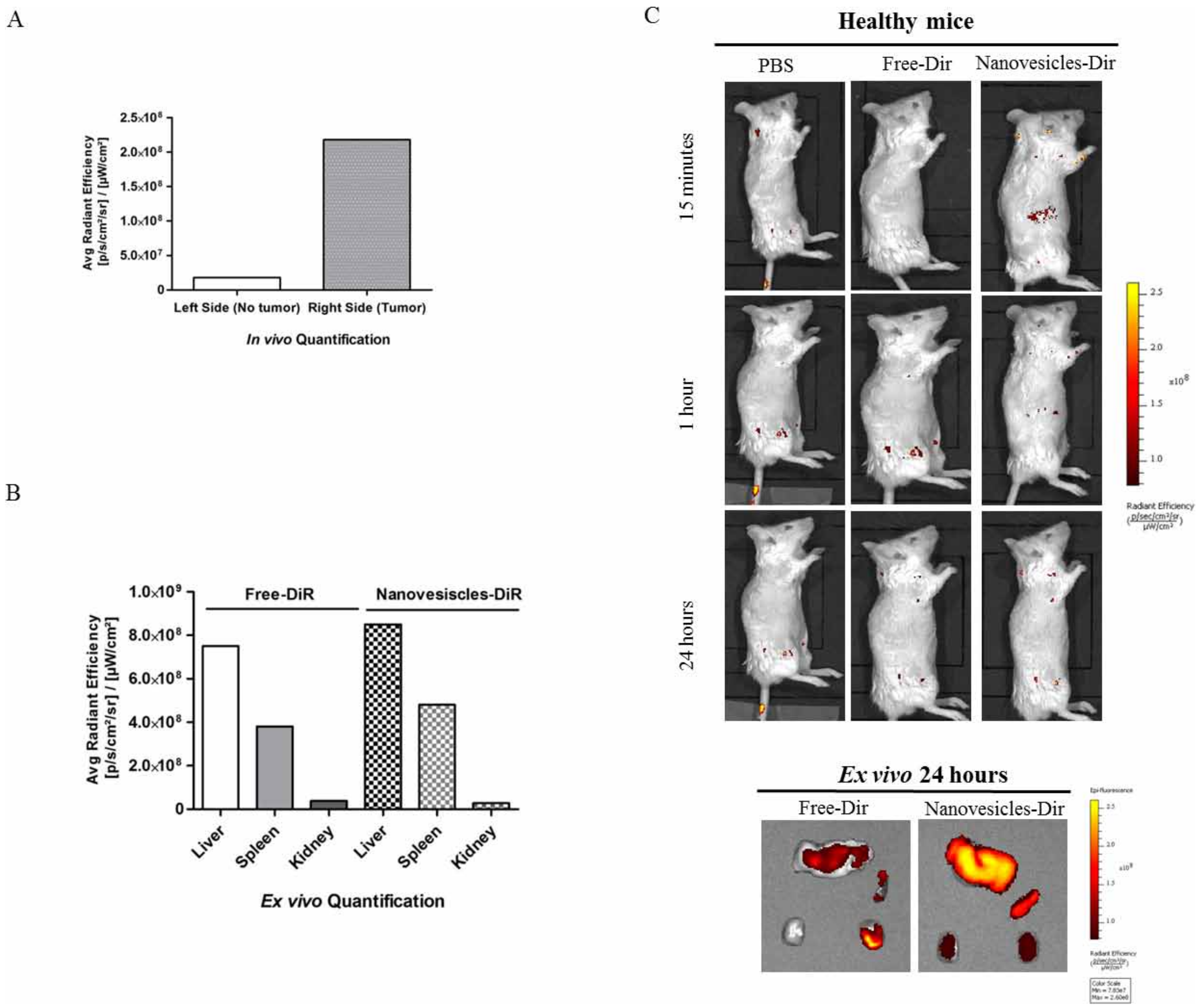

Supplementary Figure 3: A. ***In vivo*** quantification of fluorescence of left (no tumor) and right (tumor) flanks of mice bearing CML xenograft and treated with Nanovesicles-DiR. Data are expressed as average radiance efficiency ([p/s/cm^2/steradian]/[µW/cm^2]). **B.** *Ex vivo* quantification of fluorescence of organs excised after 24 h of treatment of mice bearing CML xenograft with Free-DiR or with 50 µg Nanovesicles-DiR. **C.** NOD/SCID mice were injected intraperitoneally with PBS, Free-DiR, 50 µg Nanovesicles-DiR in a volume of 150 µl PBS. Mice were imaged at 15 min, 1 h and 24 h post injection. A scale of the radiance efficiency is presented to the right of each live mouse image. Organs were excised and imaged after 24 h. A scale of the radiance efficiency is presented to the right.

Supplementary Table S1: List of *Citrus limon* L.-derived nanovesicles proteins identified by Mass Spectrometry
